# A novel questionnaire to perform teletriage of dental emergencies in children: A before-and-after study nested within a randomized clinical trial

**DOI:** 10.12688/f1000research.126388.1

**Published:** 2023-12-29

**Authors:** Ana Paula Dornellas, João Vitor Marques, Isabelle Aníbal Oliveira dos Santos, Marcelo Ramos, Júlia Mulder, Ana Estela Haddad

**Affiliations:** 1 Department of Pediatric Dentistry, School of Dentistry, University of São Paulo, São Paulo, 05508000, Brazil

**Keywords:** Teletriage, Teledentistry, Remote consultations, Pediatric dentistry, Primary teeth, Urgencies, Emergencies

## Abstract

**Background:** This will be a before-and-after study nested within a randomized clinical trial. Its objective will be to analyze the effectiveness of a teleconsultation and validate a questionnaire for performing teletriage in dental urgency/emergency situations in children aged 3 to 13, whose parents will have signed a free and informed consent form, and who have had full access to the internet.

**Methods:** The Questionnaire for Teletriage of Emergencies and Urgencies in Pediatric Dentistry (QuesT-Odontoped)—will be validated by applying it to 140 randomized child parents/guardians. After validation, another 260 children seeking emergency dental care in the municipality of Carangola, Minas Gerais, Brazil, will receive a remote consultation, be randomized, and then allocated into two groups: G1, teleconsultation, and G2, teleconsultation and face-to-face consultation (immediately after the former) with a blinded evaluator, involving anamnesis and conventional clinical examination. The G2 sample will be used in the before-after study. Both groups will be followed-up for 7 and 14 days using pain and quality-of-life scales, applied at baseline and after each follow-up period. Clinical follow-up will be carried out after 12 and 24 months to assess the outcome of the tooth that had been indicated for treatment in the teletriage. The Mann-Whitney test will be used to assess pain; Student’s t test or the Mann-Whitney test will be used to assess quality of life and the number of missing teeth after 24 months; and Poisson’s regression analysis will be used to assess the influence of other variables. The significance level will be set at 5%.

**Conclusions:** In conclusion, this study expects to confirm the hypothesis that remote urgency consultation (teletriage), through a validated questionnaire, will be able to define the planning of the clinical situation, reducing the chance of displacements and progression of infection, helping to eliminate patient pain and discomfort.

## Introduction

Pediatric patients often have difficulty communicating their pain experience. It is not uncommon for parents to become aware of the problem and seek dental care only when the pain is intense and prolonged. ^1^ Pain symptoms—whether intense, prolonged, spontaneous or nocturnal—may suggest changes in the pulp-dentin complex. Dentoalveolar trauma is common in this population, and requires immediate communication between patients and dentists, which is essential for the decision-making process involved in providing the patient with the needed first aid. ^2^ In some cases, the prognosis will depend on prompt and appropriate intervention. However, dental emergency services are not available full-time in all geographic regions of a country. In Brazil, the social isolation measures adopted by the government as a result of the COVID-19 pandemic impacted the supply and demand of dental services, and any significant delay in adopting adequate measures for cases of dental emergencies/urgencies could compromise the final results and affect the patient’s quality of life. ^3^

Within the scope of the Unified Health System (SUS), urgencies represent an important opportunity to identify the individuals and locations that are most vulnerable. Ordinance GM/MS no. 2048 of November 5, 2002, of the Ministry of Health ^4^ provides that healthcare units should carry out patient reception and risk classification screening, and that this process should be conducted by a college graduate health professional, specifically trained for conducting these procedures. The regulation also recommends that pre-established protocols must be used in order to assess the degree of urgency of each patient. Today, however, the organization of dental emergency services is still based on the rationale of order of patient arrival, contrary to the hierarchization principle adopted by the SUS, ^5^ according to which the level of care required for each case must be identified, so that access to the required services can be ensured according to their complexity. In addition, patients rely on urgent care as a gateway to the system. The Ministry of Health sought to standardize patient reception in emergency services, and standardize the care process across the country ^6^ by means of a risk classification system. By doing so, it sought to improve the work process and enhance the effectiveness of healthcare services, provided in a humanized and patient-centered fashion, with the goal of ensuring more favorable outcomes. To this end, patient reception carried out by using a risk classification system and protocol ensures proper development of work processes, and improves the resolution capacity of health services. In addition, this system and protocol ensure prioritization of the more severe clinical cases, and an appointment scheduling based on patient demands. ^7^ Studies in the literature have used questionnaires to diagnose dental problems in children, such as caries lesions, dental calculus, gingivitis, dental fractures, and malocclusions. ^8^^,^^9^ However, to the best of our knowledge, none have proposed a model questionnaire for dental urgency cases, providing guidance for the proper referral of these patients.

Faced with the COVID-19 pandemic, each country has adopted different policies to resume its dental care services. The United Kingdom, for instance, chose to select cases through teledentistry, and to see only those patients requiring urgent treatment. ^10^ In Brazil, Resolution 226 of June 4, 2020, ^11^ and Ordinance No. 526 of June 24, 2020 ^12^ of the Ministry of Health have regulated teleconsultation, telemonitoring, and teletriage procedures for dentists working in primary care. In this respect, teledentistry is a method of proven capability to provide healthcare to underserved population groups, or to those unable to attend a face-to-face consultation. ^13^^‒^^16^ Teledentistry may also be a viable alternative for assessing deferrable and non-deferrable urgency cases, by conducting a screening process based on available information and communication technologies, e. g. video calls or text messages containing photos. ^17^^‒^^21^ The World Health Organization (WHO) ^22^ recommended that its member-countries make use of telehealth as a strategy to improve the quality of services provided, even before the pandemic. ^23^

It is known that teledentistry has been considered a practical and economically viable strategy to provide healthcare to underserved populations, including the socially disadvantaged, those living in remote locations or rural areas, or those who simply do not have access to routine dental care. ^24^^‒^^28^ In this context, teletriage has been associated with streamlined patient referral, shorter waiting lines, and improved ordering of care priorities. Based on an accurate assessment provided by remote screening, patients can receive proper primary support in an appropriate and customized fashion, and be directed to telecare or to the dental office itself, when needed, thus minimizing unnecessary displacements and contamination risks. ^29^ However, consistent scientific evidence is still lacking on the advantages of teletriage in dentistry; therefore, further investigation into the possibilities of using telecommunication tools in the planning of health actions is warranted, particularly for the sake of municipalities that do not have the human resources to provide specialized care, and general populations that do not have access to a specialist.

### Objectives

The foremost aim of this study will be to analyze the effectiveness of teletriage and of an urgency/emergency risk classification system for children, compared to face-to-face consultation for these patients. Secondarily, other key issues will be investigated, as follows:

1 - validation of a questionnaire for dental urgency screening in children;
2 - assessment of the level of agreement among diagnoses and risk ratings assigned to cases screened in person versus remotely;
3 - proposal and testing of an oral health protocol for patient reception and risk classification for children seeking an urgency/emergency dental care service.

## Protocol

### Ethics statement

The design of this study (both before-and-after study as well as the randomized clinical trial) was approved by the Research Ethics Committee, School of Dentistry, University of São Paulo (approval no. 46974821.9.0000.0075), on June 1st, 2021. It was also submitted to *The Brazilian Clinical Trials Registry*, code RBR-523hrsx. The research protocol was written following SPIRIT *(Standard Protocol Items: Recommendations for Interventional Trials)* guidelines. ^30^

The study will be divided into the following stages: (1) questionnaire validation, (2) randomized clinical trial, and (3) before-and-after study, each with their own methodology.

*Team and participants*

The allocation sequence was generated by an external professional (Dentist, PhD);

The participants enrollment and their assignment to interventions will be carried out by the main researcher (APD).

*Questionnaire validation*

The inclusion of the research participants in this stage will be made upon a digital acceptance of a free and informed digital consent form (FICF), which will be digitally signed by the person in charge of the child (parents/guardians) and uploaded through a digital link, for this remote part of the study. This form presents the research objectives clearly, as well as the guidelines that will be provided to patients. Permission to disclose data will be part of this document, and shall ensure confidentiality of participant identity. All the participants will be informed that their participation in the study is voluntary and the participants will be informed that their anonymised data will be published.

*Before-and-after study nested within a randomized clinical trial*

For this stage, inclusion of participants in the study groups will also take place upon a digital acceptance of a free and informed digital consent form (FICF), uploaded through a digital link, and parents or guardians must mark the tick – box to consent for the remote part of the study, on behalf of the child. The parents/guardians of participants assigned to the face-to-face consultation group (G2) will also be required to sign a FICF.

**Figure 1.**
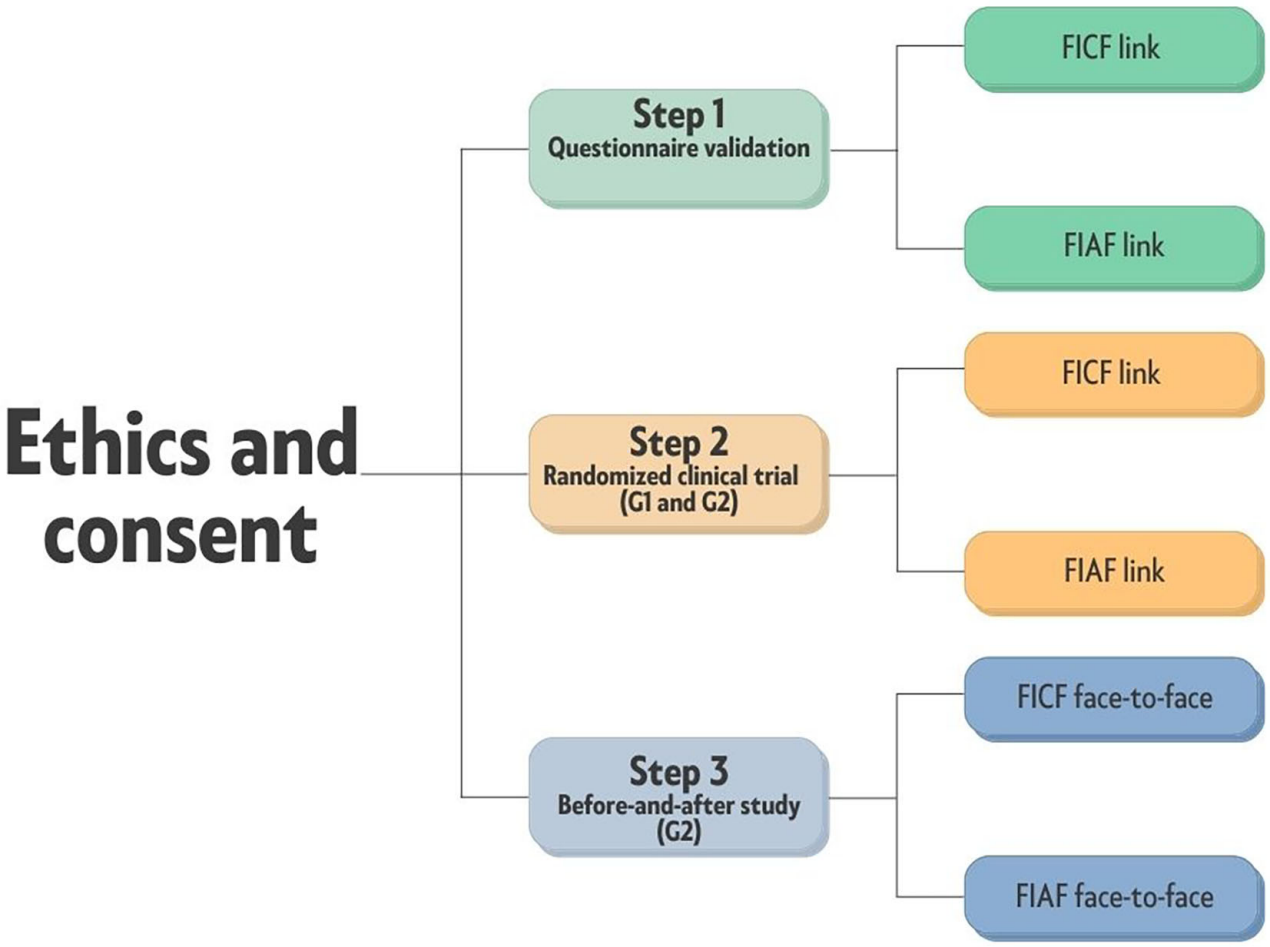
Application of the FICF (free and informed consent form) to parents/guardians and FIAF (free and informed agreement form) that will be provided to the children. G1, teleconsultation; G2, teleconsultation plus face-to-face consultation.

In both stages (questionnaire validation and before-and-after study nested within a randomized clinical trial), in addition to the parent/guardian consent, the study will consider the age and level of understanding of the participants in the sample, whose agreement to participate will be obtained by reading a specific document, or verbally for those who cannot read and/or access the link provided. The form of acceptance will depend on the child’s maturity level, which may only be verbal, which does not necessarily require a signature, according to the Ethic Committee guidelines and approval. Children belonging to G2 (in person) and with sufficient understanding will be invited to fill out, in person, the same free and informed agreement form (FIAF) that had been sent to their parent/guardian online in stage 1, totaling two filled-out forms for G2 ( Figure 1). In this case, parents/guardians are also required to fill out an informed agreement.

### Confidentiality

The data collected will be stored using specific procedures to ensure the secrecy and confidentiality of the participants’ information. The access to the final trial dataset will be exclusive to the main researcher and the project supervisor. Only remote consultation platforms that have robust security standards, and that meet the regulations of the General Data Protection Law (LGPD) will be used to store the data. In addition, the research subjects will have the right to leave the study at any time, with no detriment to their care.

### Research design

*Questionnaire validation*

**Figure 2.**
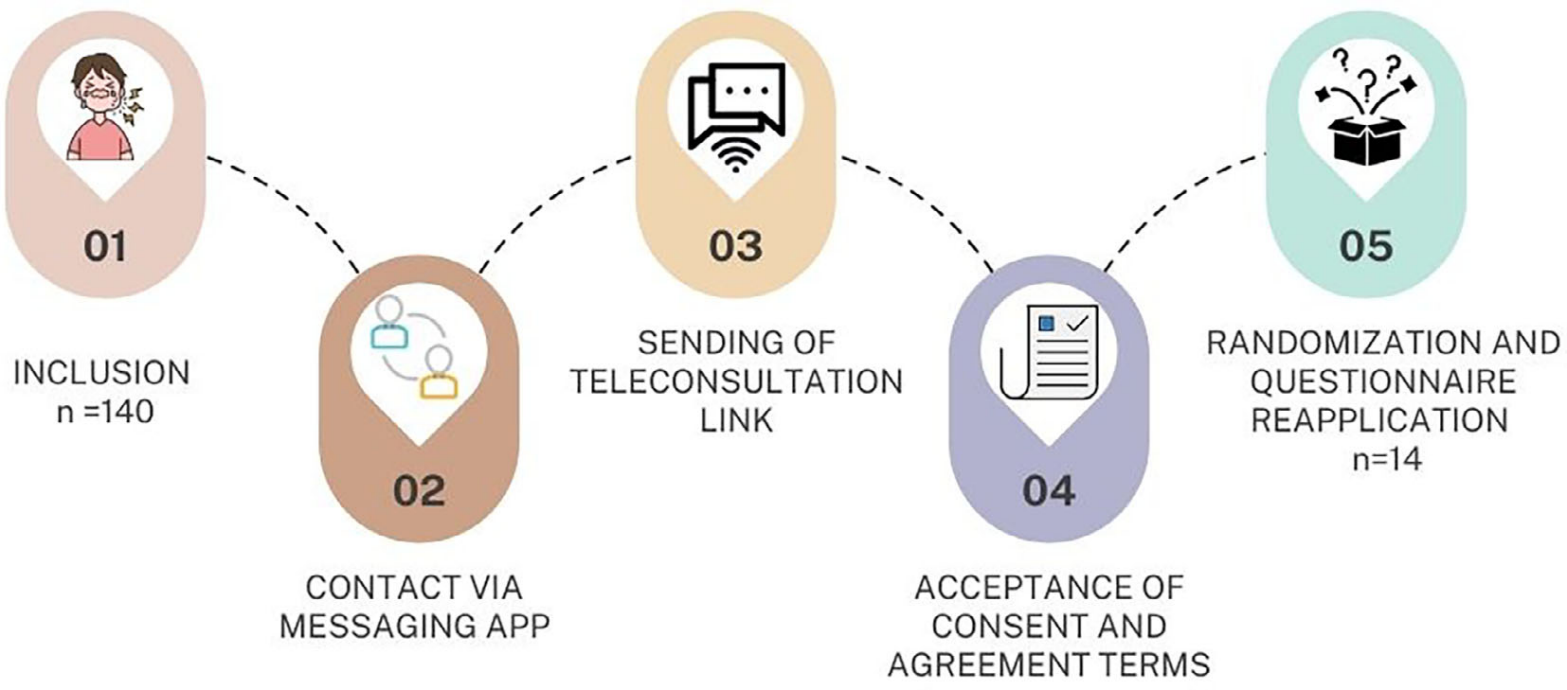
Validation of the questionnaire applied in the study.

The parents/guardians of children with dental pain who seek care at the University of São Paulo School of Dentistry or the University of São Paulo Health Superintendence (SAU) will receive the contact information for one of the researchers, complete with a telephone number, and information about the requirements for having a remote consultation. The questionnaire will be applied to 140 participants, and reapplied to 14 randomized participants ( Figure 2). Randomization, in blocks of four participants, will be performed using freely available statistical software ( MedCalc ^®^ 15.11, MedCalc Software, Ostend, Belgium), and the sequence generated will be distributed in sealed brown envelopes. The envelope will only be opened after the patient accepts the FICF or FIAF at the time of the teleconsultation performed via Video For Health (V4H) platform, a service dedicated to digital health with a video call management functionality and aligned with the LGPD in Brazil.

*Before-and-after study nested within a randomized clinical trial*

**Figure 3.**
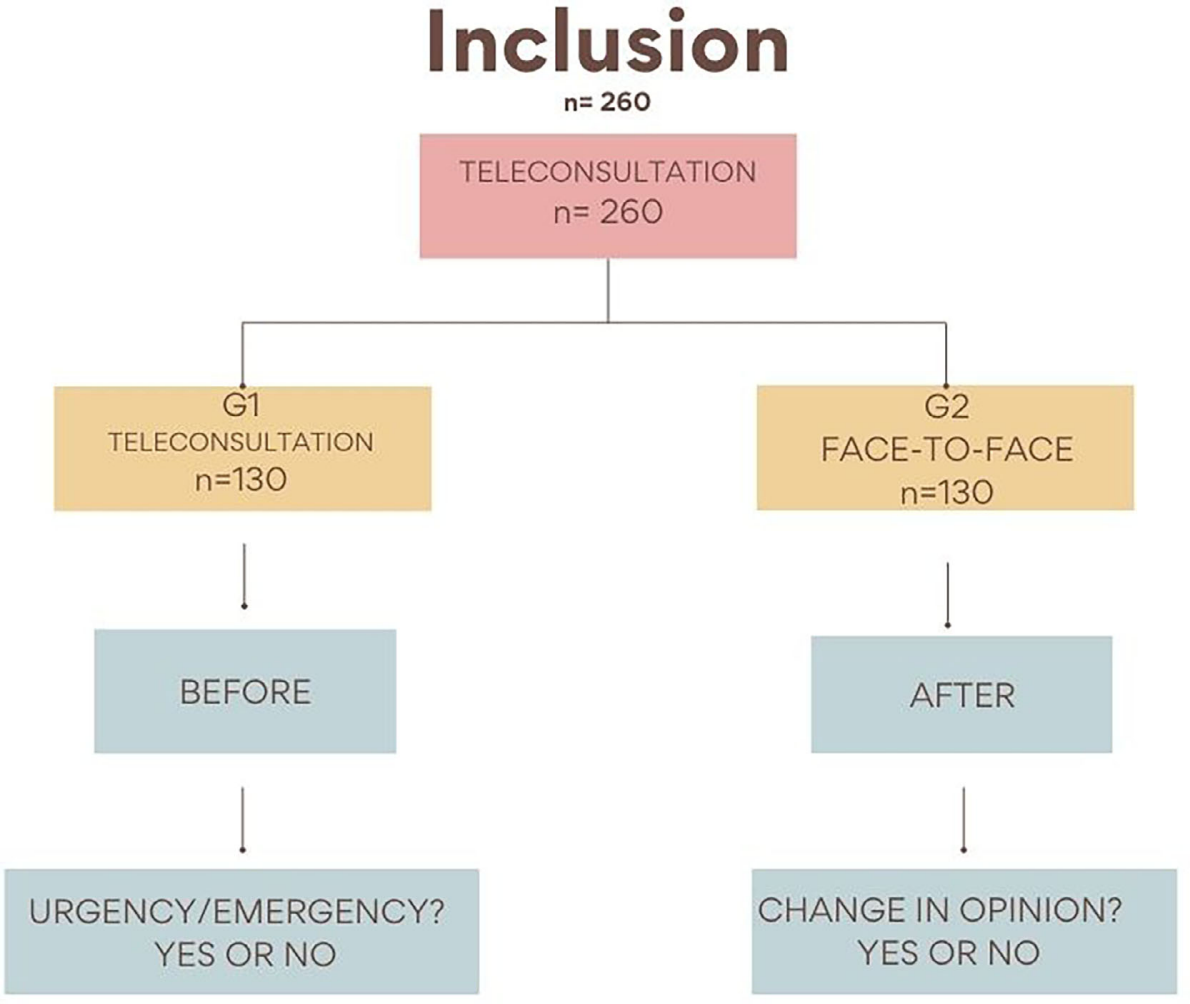
Inclusion of patients in the randomized control trial (before and after). G1, teleconsultation; G2, teleconsultation plus face-to-face consultation.

The present research project will consist of a randomized, controlled, noninferiority, and blinded (examiners) clinical study, with 260 children aged 3 to 13 years in dental urgency situations. All of the participants investigated will be users of Basic Health Units, hospitals or dental clinics, residing in the municipality of Carangola, MG, Brazil. A link providing access to the Telehealth and Teledentistry Center from the University of São Paulo School of Dentistry (Nutes/FOUSP) platform will be sent to each patient after said patient makes the first contact through *WhatsApp* , a messaging app. All patients will be previously evaluated by a previously trained researcher in a teleconsultation (“before”), and then randomized and divided into two groups: G1, comprising 130 patients seen via a teleconsultation, and G2, comprising 130 patients seen via a teleconsultation and also in person ( Figure 3). The face-to-face evaluation (“after”) will be performed by two researchers who will be blinded to the result of the teleconsultation, who will perform anamnesis and clinical examination using a specific dental record, and who will answer whether or not the patient’s case is an urgent situation at the end of the evaluation. The dental condition motivating inclusion of the patient in the study will be treated by the researcher, and any other patient needs will be seen to by referring them to the health unit where they were registered.

**Figure 4.**
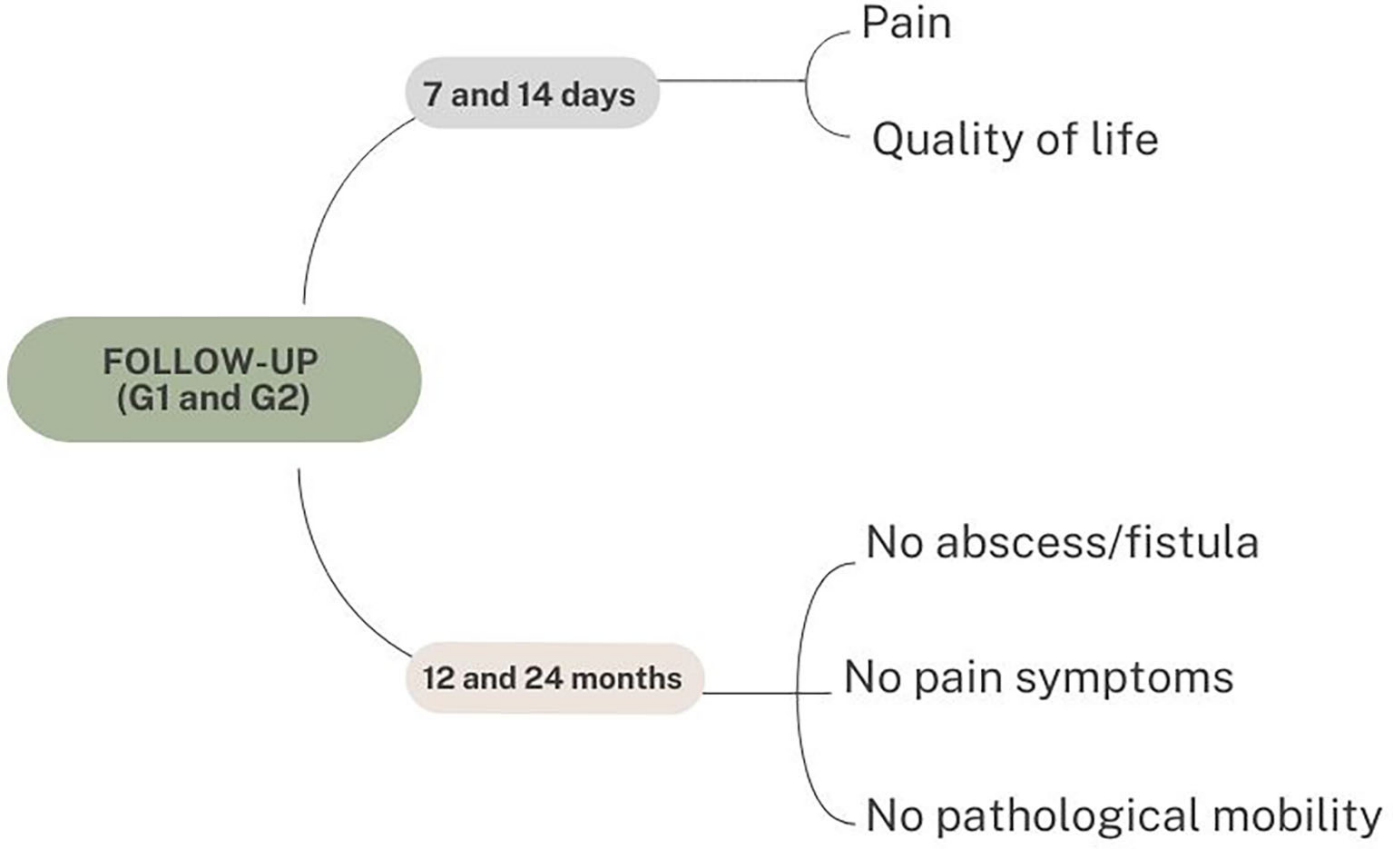
The clinical criteria used to determine treatment success during patient follow-up. G1, teleconsultation; G2, teleconsultation plus face-to-face consultation.

Patients in G1 and G2 will be followed up for seven and 14 days using specific instruments to assess the absence or presence of pain (Wong-Baker scale) ^31^ and quality of life, according to the child’s age (Child Perceptions Questionnaire, CPQ, ^32^ or Early Childhood Oral Health Impact Scale, ECOHIS). ^33^ In the case of urgencies involving teeth, a 12- and 24-month follow-up of the tooth reported in the teletriage will be carried out. The clinical criteria used to determine treatment success will be no fistula or exudate, no abscess, no painful symptoms, and no pathological mobility ( Figure 4).

### Inclusion and exclusion criteria

The inclusion criteria will be the same for the two stages of patient selection in the study.

*Inclusion criteria*

a) children aged 3 to 13 years, with dental pain, whose parents seek urgent care at the Dental School of the University of São Paulo in São Paulo, or at the public network in the city of Carangola, in the state of Minas Gerais.
b) patients with a sufficiently good internet connection to allow a synchronous consultation.

*Exclusion criteria*

The exclusion criteria will be the same for the two stages of patient selection of the study:

a) patients who fail to attend the face-to-face consultation, when assigned to G2;
b) patients with internet access difficulties.

### Recruitment

*Questionnaire validation stage*

The parents/guardians of children with dental pain who seek care at the University of São Paulo School of Dentistry or at the University of São Paulo Health Superintendence (SAU) will receive the contact information for one of the researchers, complete with a telephone number, and information about the requirements for having a remote consultation.

*Before-and-after study nested within a randomized clinical trial*

When arriving at a Basic Health Unit, a hospital or a dental clinic outside opening hours, the parents/guardians of children with dental pain will find a poster outside containing the contact information for one of the researchers, complete with her/his telephone number, the ethics committee approval number, and the requirements for having a remote consultation.

### Assignment

*Questionnaire validation*

The questionnaire will be applied to 140 participants, and reapplied to 14 randomized participants. Randomization, in blocks of four participants, will be performed using statistical software (MedCalc ^®^ 15.11, MedCalc Software, Ostend, Belgium), and the sequence generated will be distributed in sealed brown envelopes.

*Before-and-after study nested within a randomized clinical trial*

Randomization, in blocks of four patients, to assign them to one of the treatment groups, will be performed using statistical software (MedCalc ^®^ 15.11, MedCalc Software, Ostend, Belgium), and the sequence generated will be distributed in sealed brown envelopes. The envelopes must be opened immediately after the start of the teleconsultation.

### Training of examiners/researchers

The examiners/researchers (authors on the paper and two dental practitioners based at the clinics) will be trained by the author responsible for the project (APD) to carry out the research prior to the first stage. This will involve a four hour training program for urgency situations of dental origin in children, and a 4-hour calibration program for questionnaire application.

Prior to the beginning of the study, the researcher will explain the study and how teledentistry will be used within the Family Health Units to the entire team.

### Interventions

The synchronous teletriage will be carried out using the teleconsultation-dedicated NuTes-FOUSP platform that complies with the LGPD, as well as a questionnaire containing objective questions to ascertain whether or not the situation in question is a case of a dental urgency. The time required to apply the questionnaire will depend on how long it takes to obtain the answers from the patients’ parents/guardians.

In the clinical stage of the study, the time of the face-to-face dental consultation, to be performed immediately after the synchronous consultation shall not exceed one hour.

The clinical situations listed below will be considered dental urgencies (ADA, 2020) ^2^:

• irreversible pulpitis;
• pericoronitis;
• abscess or localized bacterial infection, resulting in localized pain and swelling;
• tooth fracture, resulting in pain or causing soft tissue trauma.
• dental trauma, involving avulsion and/or luxation;
• a missing or fractured restoration, or one causing gingival irritation and requiring a provisional restoration;
• extensive caries or a defective restoration that is causing pain;
• pain that requires replacing the provisional filling of an endodontic access opening;
• perforated or ulcerated oral mucosa, requiring trimming or adjustment of a wire or orthodontic appliance.

The clinical situations listed below will not be considered dental urgencies:

• initial or follow-up dental examination;
• routine radiography;
• dental prophylaxis;
• routine periodontal therapy;
• orthodontic procedure other than that needed to treat an acute problem (e.g., pain, infection, trauma);
• extraction of an asymptomatic tooth;
• restorative dentistry procedure, including treatment of an asymptomatic carious lesion;
• aesthetic dental procedure

### Complications

*Questionnaire validation*

The risks involved in this study are those inherent in virtual environments, electronic media, or non-face-to-face activities, considering the limitations of the technologies used or those of researchers in ensuring data confidentiality and preventing data violation.

*Before-and-after study nested within a randomized clinical trial*

In addition to the risks mentioned above, there are other risks inherent in any pediatric dental treatment, including those related to the possible failure of the proposed dental treatment. Additional risks are those related to data collection, such as possible discomfort felt during the remote and/or face-to-face consultation, during the assessment, examination, and therapeutic procedures themselves, and possible annoyance and/or emotional or social stress occasionally felt by the patient and/or by parent/guardian when answering questions related to the child’s health.

To reduce these risks, the procedures will be monitored by the researcher, who will be prepared to provide all the assistance needed. In the event of personal injury caused directly by any procedure or treatment proposed in this study, the child will be entitled to free dental treatment.

### Data collection

The data will be collected at different timepoints, as shown in Table 1.

**Table 1.** Data collection. G1, teleconsultation; G2, teleconsultation plus face-to-face consultation.

	Time +/ -	Timepoint	Research actions
Training of examiners/researchers	8 hours	t-1	The examiners/researchers will be trained to carry out the study, including a 4-hour training program for urgencies of dental origin in children, and a 4-hour calibration program for questionnaire application.
Inclusion criteria - Quest - Odontoped	12 months	0	The parents/guardians of children with dental pain, who seek care at the University of São Paulo School of Dentistry, or at the University of São Paulo Health Superintendence (SAU), will receive the contact information for one of the researchers, complete with a telephone number, and information on the requirements for having a remote consultation.
Inclusion criteria (test and control groups) - Carangola, MG, Brazil	12 months	0	When arriving at a Basic Health Unit, a hospital or a dental clinic outside opening hours, the parents/guardians of children with dental pain will find a poster outside containing the contact information for one of the researchers, complete with a telephone number, the number of the ethics committee approval report, and the requirements for having a remote consultation.
Follow-up	1 week and 2 weeks	T1	Patients in G1 and G2 will be followed-up for 7 and 14 days using specific instruments to assess the absence or presence of pain (Wong-Baker scale), and the quality of life according to the child's age.
Follow-up	12 months and 24 months	T2	In the event of urgencies involving teeth, a 12- and 24-month follow-up of the tooth reported in the teletriage will be carried out. The clinical criteria used to determine treatment success will be no fistula or exudate, no abscess, no painful symptoms, and no pathological mobility.
Close-out	24 months	tx	Analysis and dissemination of research results.

### Sample size

*Sample for questionnaire validation*

The guidelines of the European Statistical System stipulate that the development and validation of instruments must follow five steps: (1) conceptualization, (2) questionnaire design, (3) questionnaire testing, (4) revision, and (5) data collection. ^34^ Considering that the questionnaire conceptualization and design have already been completed, our considerations will begin from the questionnaire testing step. The first step involved in instrument testing is evaluation by a panel of experts (content validation). There is a controversy in the literature regarding the recommended size for this panel, ranging from five to twenty members, ^35^^,^^36^ depending on their experience and qualifications. Subsequently, data collection will be carried out by applying the questionnaire (criterion validity). To this end, a sample size of at least 10 participants is recommended for each item ^37^; therefore, the minimum sample size of the present study will be 140 subjects. In order for the instrument to be considered reliable (test and retest), it must be able to produce similar results when applied to the same individual at different times, and by different interviewers. For this purpose, a sample size corresponding to 10% of the total “N” (i.e., 14 patients in the current version of the questionnaire) should be used.

*Sample size calculation for the RCT*

Primary outcome: the presence of dental pain in children and adolescents, considering independent samples (in this case, there is no pairing of individuals, and the interventions will be assigned to different individuals). The possibility of dropouts during the follow-up period was taken into account.

In performing the sample size calculation for independent samples, the proportion of dental pain observed in children and adolescents will be considered as 32.7%, ^38^ and the minimum, clinically relevant proportion difference will be 15%. Thus, considering a two-tailed test, a significance level of 0.05, and a power of 0.80, the calculation will yield a total number of 236 patients. Owing to the possibility of participant dropout during the follow-up period, 10% of this number will be added to the final sample, bringing the total number of patients required per experimental group to 130 (G Power, version 3.1.9.4).

*Sample size calculation for the before-and-after study*

Before-and-after studies are considered observational studies, and use a preliminary diagnostic test to decide whether or not to treat a series of examined patients. These patients are submitted to another diagnostic method, and the treatment decision can then be revised. Since this before-and-after study is nested within a randomized controlled trial (RCT), the sample size calculation will take into account the primary outcome of the RCT and the two experimental groups. However, only the group evaluated using both methods (teletriage + face-to-face consultation) will be eligible for this study. Therefore, the before-and-after study sample will consist of 130 patients.

*Outcomes*

Primary outcome: the presence of dental pain in children and adolescents, considering independent samples.

Secondary outcome: quality of life (questionnaires according to age group) and tooth loss.

### Data analysis

*Questionnaire validation*

The kappa coefficient (k) and content validity index (CVI) will be used to quantify the degree of agreement among experts during content validation. In order to assess the reliability of the instrument, the test and retest results will be evaluated using the intra-examiner agreement test (kappa test, *k*), which is the appropriate statistical procedure to assess the reliability of categorical and nominal variables. The criterion validity will be analyzed by comparing the results of the teleconsultation with those of the face-to-face consultation using the chi-square test (χ ^2^). The significance level will be set at 5%, and the data will be analyzed using Jamovi and RStudio software.

*Randomized clinical trial*

Primary outcome: pain (Wong-Baker scale); secondary outcomes: quality of life (questionnaires according to age group) and missing teeth.

The primary outcome of the randomized clinical study will be any change in pain score observed at the 7-day and 14-day follow-up timepoints compared to baseline. This outcome comprises an ordinal qualitative variable; therefore, comparison between the teleconsultation and face-to-face consultation groups will be performed using the Mann-Whitney test.

Other secondary outcomes will be evaluated, such as the impact of treatments on oral health-related quality of life, and the number of missing teeth after the 24-month follow-up period. Differences between the groups regarding the final and initial scores of the questionnaires on oral health-related quality of life, as well as the number of missing teeth, will be compared using Student’s t test or the Mann-Whitney test, depending on the type of data distribution (normal or non-normal). In addition, Poisson’s regression analysis will be performed to assess the influence of other variables on the results. The significance level will be set at 5%, and the data will be analyzed using Jamovi and RStudio software.

*Before-after study nested within an RCT*

Descriptive analyses regarding diagnosis for needing urgent treatment will be performed using the teletriage questionnaire, alone and in combination with the face-to-face consultation. The possible treatment decision outcomes for these analyses, and for both the remote and the face-to-face examination, will be (i) the need for urgent treatment or (ii) the possibility of elective treatment. The frequency of change in the treatment decision will be calculated considering that the change can be (i) from urgent to elective treatment or (ii) from elective to urgent treatment. Explanatory variables related to the children, such as sex, age (randomization strata), and caries experience may be used in the analyses. The primary outcome in this study will be any change in treatment decision after the face-to-face consultation. Prevalence ratio (PR) values and respective 95% confidence intervals (95% CI) will be calculated, and univariate and multiple regression analyses will be performed. The significance level will be set at 5%, and the data will be analyzed using Jamovi and RStudio software.

### Study status

The study is in the phase of inclusion of patients; and the questionnaire is in the validation stage. Completion of this project is scheduled for the second half of 2024.

### Dissemination

The results of this study will be presented at scientific meetings and published in peer-reviewed medical journals.

## Conclusions

The hypothesis of the present study is that the synchronous remote urgent consultation (or teleconsultation) performed using a validated questionnaire will be able to determine the appropriate screening decision for the clinical situation in which the patient finds him/herself.

## Data Availability

No data are associated with this article.
