# Supplementary material for: A novel questionnaire to perform teletriage of dental emergencies in children: A before-and-after study nested within a randomized clinical trial
Source: F1000Res. 2023 Dec 29;12:1610. [Version 1] doi: 10.12688/f1000research.126388.1 (PMC11150902; doi:10.12688/f1000research.126388.1)
Supplement: Peer review history

# Reviewer report: Reviewer response for version 1

**DOI:** 10.5256/f1000research.138795.r278729

**Published:** 2024-06-04

**Authors:** Alexandre Baudet

**Affiliations:** 1 Universite de Lorraine, Nancy, Grand Est, France

This study protocol presents the rationale and describes the methodology used to analyze the effectiveness of a teleconsultation and validate a questionnaire for performing teletriage in dental urgency/emergency situations in children aged 3 to 13 in Brazil. A before-and-after study nested within a randomized clinical trial will be carried out with 130 children in the group G1: teleconsultation only (control group) and 130 children in the group G2: teleconsultation plus face-to-face consultation (before-after group). The dental pain and the quality of life assessed with questionnaires according to age group will be the main outcomes with a following time of 24 months.

 The paper is well-structured and easy to follow. One comment for the authors to consider: figure 3 is unclear and not in accordance with the text, it should highlight that G1 = teleconsultation only (control group), G2 = teleconsultation (before) plus face-to-face consultation (after); actually G1 is indicated as before period and G2 as after period.

Is the study design appropriate for the research question?

Yes

Is the rationale for, and objectives of, the study clearly described?

Yes

Are sufficient details of the methods provided to allow replication by others?

Yes

Are the datasets clearly presented in a useable and accessible format?

Not applicable

Reviewer Expertise:

Dentistry; Public health; Infection prevention and control; Antibiotic stewardship, Antibiotic resistance

I confirm that I have read this submission and believe that I have an appropriate level of expertise to confirm that it is of an acceptable scientific standard.

# Reviewer report: Reviewer response for version 1

**DOI:** 10.5256/f1000research.138795.r265384

**Published:** 2024-05-15

**Authors:** Shimaa Kotb

**Affiliations:** 1 Sphinx University, New Assuit, Egypt

Breif Description of Article: Teledentistry article show the importance of the remote delivery of clinical care through electronic communications. Dental’s virtual consultation is a convenient way to connect with patients from the comfort of their homes to show support and interest in their oral health state .

 Strength: Article is interesting, Informative, Discuss helpful effective tools in planning the treatment and screening patients easily without time consuming, Effective method for reassurance patient in emergency situation and in alleviate the sever pain.

 Weakness: 1.Need more research in the future on this issue with large sample size. 2. Some References need to be updated

 Summary: Teledentistry, a promising tool, is still not widely used in dentistry. Teledentistry enables remote assessment, allowing for efficient screening and triage of patients. Teledentistry could be as effective as face-to-face technology for oral screening, particularly in school-based programs, caries assessment, referrals, and teleconsultations, especially during emergencies.

 Decision : Accepted with minor correction (related to the update references)

Is the study design appropriate for the research question?

Yes

Is the rationale for, and objectives of, the study clearly described?

Yes

Are sufficient details of the methods provided to allow replication by others?

Yes

Are the datasets clearly presented in a useable and accessible format?

Not applicable

Reviewer Expertise:

Oral medicine ,oral pathology ,periodontology ,dental implants .dental surgery ,Dental Radiology .

I confirm that I have read this submission and believe that I have an appropriate level of expertise to confirm that it is of an acceptable scientific standard.

## References

[1] : A novel questionnaire to perform teletriage of dental emergencies in children: A before-and-after study nested within a randomized clinical trial. *F1000Research* .2023;12: 10.12688/f1000research.126388.1 10.12688/f1000research.126388.1 37476505
